# Capnography-guided awake nasal intubation in a 4-month infant with Pierre Robin syndrome

**DOI:** 10.4103/0019-5049.65349

**Published:** 2010

**Authors:** Rajeev Sharma

**Affiliations:** Department of Anaesthesia, ESI Hospital, Rohini, New Delhi, India

Sir,

I read the case report by Dr. Patra with interest.[1] I congratulate the author for his innovative technique that achieved an awake blind nasal intubation in an infant with Pierre Robin syndrome after previously failed intubation attempts. Having used capnography as an aid to intubation,[2–4] I have the following reservations against this technique which need to be highlighted.

First, the technique involves introduction of sampling tube of a capnograph through the tracheal tube. The male connector at the sampling end needed to be sacrificed for this purpose. This necessitates the need for a spare sampling tube that would result in an increase in the cost.

Second, the technique includes taping the sampling tube to the tube connector. Therefore, one has to be very careful so as to prevent kinking of the sampling tube during intubation attempts. This may necessitate the help of an assistant to stabilize the tubing especially at the take-off point.

Third, when introducing the tracheal tube through the nose, the anaesthetists assume that the tip of the EtCO_2_ tubing is patent. However, during manipulation of the tracheal tube, chances of the sampling tube tip abutting against the wall of tracheal tube cannot be completely ruled out. This could give false negative results, as far as correct placement of the tracheal tube is concerned.

Fourth, after getting a regular capnogram, one needs to remove the tubing from the lumen of the tracheal tube and confirm by manual ventilation. However, one must remember that further intubation attempts may still be required (e.g. in cases of failure of the initial attempt or dislodgement of tracheal tube while withdrawing the sampling tube which involves removal of the adhesive tapes at the machine end). In this clinical setting, this will mandate complete removal of the nasal tube outside, preparation of the assembly again and repetition of the whole procedure. This may not be acceptable in difficult cases because of additional time wastage and the potential for trauma during every repeated attempt of nasal tube insertion.

**Figure 1:**
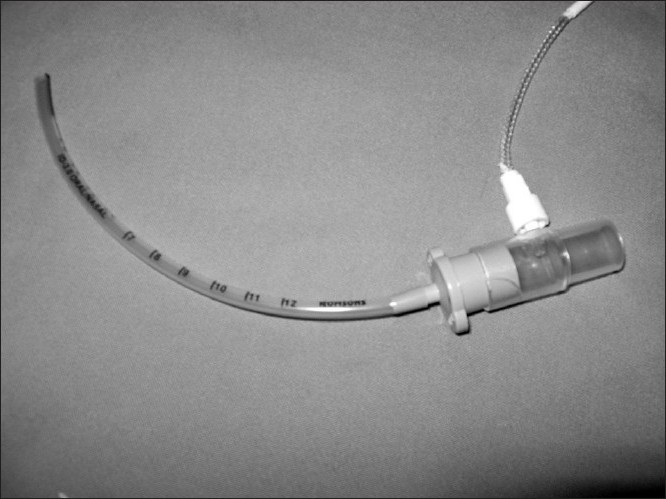
The connector of a EtCO_2_ can be directly connected to the machine end of the tracheal tube. This is a simpler, more effective, less time consuming, cheaper and reproducible technique using the available equipment without any added wastage

Further, I would like to mention a much simpler, more effective, less time consuming, cheaper and reproducible technique using the available equipment without any added wastage. This technique involves connecting the EtCO_2_ tubing with a connector directly over the machine end of the tracheal tube connector [Figure 1]. This circumvents many of the shortcomings of the author’s technique.
